# Phylogenetic study of the endemic species *Oxytropis almaatensis* (Fabaceae) based on nuclear ribosomal DNA ITS sequences

**DOI:** 10.1186/s12870-017-1128-x

**Published:** 2017-11-14

**Authors:** Shyryn Almerekova, Nashtay Mukhitdinov, Saule Abugalieva

**Affiliations:** 1 Institute of Plant Biology and Biotechnology, Almaty, Kazakhstan 050040; 2 al-Farabi Kazakh National University, Department of Biodiversity and Bioresources, Almaty, Kazakhstan 050040

**Keywords:** Fabaceae, *Oxytropis*, *Oxytropis almaatensis*, Its, Phylogeny, Taxonomy

## Abstract

**Background:**

*Oxytropis almaatensis* Bajt. is a rare, narrow endemic species of the Trans-Ili Alatau mountains in Kazakhstan. Up to now, no studies regarding the taxonomy and variation of key morphological traits of *O. almaatensis* were undertaken. The purpose of this analysis was to evaluate phenotypic variation of *O. almaatensis* and assess the position of the species within the genus based on nucleotide sequences of the nuclear ribosomal DNA internal transcribed spacer (ITS) region.

**Results:**

Two populations of *O. almaatensis* were collected in neighboring gorges of the Trans-Ili Alatau Mountains. The ITS sequences from the samples of two populations of *O. almaatensis* were identical. The phylogenetic analysis indicated that *O. almaatensis* is within *Oxytropis* genetically close to *O. glabra* as these species formed a separate subclade. The phenotypic variation of populations was assessed using nine morphological traits and compared to descriptions of *O. glabra*. The range of variation for the traits between two populations was established. A clear morphological difference of *O. almaatensis* and *O. glabra* was found in peduncle length to leaf length ratio. This was in *O. almaatensis* 1.56, while in *O. glabra*, it was 1.0*.*

**Conclusions:**

The study provides the first phenotypic description and phylogenetic placement of the rare endemic species *O. almaatensis*. The morphological traits in two *O. almaatensis* populations showed a high level of phenotypic variability. Although clearly different from *O. glabra*, the ITS phylogeny grouped these species in a subclade within the genus.

**Electronic supplementary material:**

The online version of this article (10.1186/s12870-017-1128-x) contains supplementary material, which is available to authorized users.

## Background

The genus *Oxytropis* DC. is part of the Fabaceae family, and occurs with 450 species world-wide [1]. It is very diverse in Central Asian mountain regions [2]. The genus is part of the tribe *Galegeae* and traditionally grouped with *Astragalus* [3]. *Oxytropis* was originally considered as a part of *Astragalus* by Linnaeus, but was recognized as a distinct genus by De Candole (1802) who separated *Oxytropis* based on keel petal shape (pointed in *Oxytropis* vs. obtuse in *Astragalus*) and pod septum shape (arising from adaxial suture in *Oxytropis* vs. abaxial in *Astragalus*) [4].

*Oxytropis* is one of the largest genera in the Central Asian flora [2]. Several studies based on molecular data reported that *Oxytropis* and *Astragalus* are sister groups [5, 6], and diverged approximately 12–16 Ma ago [7]. In the northern Tian-Shan, *Oxytropis* has been studied by Abdulina [8]. According to Abdulina [8], the distinguishing traits in *Oxytropis* are life form, stem development level, the level of the outgrowth of stipule with stem and each other, shape and structure of the beans, shapes and sizes of the corolla and calyx, and flower structure. Based on Malyshev’s taxonomy [1], *Oxytropis*, in Asian-Russia, consists of 6 subgenera and 25 sections. Hence, *Oxytropis* is one of the most complex and polymorphic genera in Fabaceae. Thus, there have been a number of studies to clarify the phylogenetic relationship among species of the genus. For instance, thirteen native *Oxytropis* species in Turkey were investigated to identify the phylogenetic relationships using the *trn*L intron, *trn*L-F intergenic spacer, and *trn*V intron of chloroplast (cp) DNA. Within the studied regions, the *trn*L intron was the most variable of the three regions studied [9]. In Asian Russia, several species from subgenera *Oxytropis* and *Phacoxytropis* of *Oxytropis* were studied utilizing the *trn*H*–psb*A*, trn*L*–trn*F *and trn*S*–trn*G intergenic spacer regions of cpDNA [9]. Despite these reports and a number of other publications [10–13], a survey of scientific literature suggests that there is no universally accepted taxonomy for the genus [14]. Although general boundaries for subgenera and sections of the genus are broadly agreed among taxonomist from different continents, contradictory results in taxonomy can be explained by several factors: mis-identified samples, inaccurate sequencing, or different ploidy level of species within the genus [15].

*Oxytropis* is diverse and rich in endemism, and represented by 153 species in Central Asia [2]. In Kazakhstan, the genus comprises 119 species, 36 of which are endemic [16], and 10 are listed as endangered [17]. One of those endemic species, *Oxytropis almaatensis* Bajt., has been reported as a rare and narrowly endemic species growing in the Turgen Gorge of Trans-Ili Alatau mountains [8, 18, 16] and potentially is an important medicinal plant [19]. There are several reports suggested that the species belongs to the section *Eumorpha*. Morphologically, the species is similar to *O. glabra* [18], except *O. glabra* belongs to section *Mesogeae* and has a tall stem, while *O. almaatensis* is a stemless plant.

Despite the richness and specificity of the flora of Kazakhstan, there are only few examples of the evaluation of wild plant species using genetic markers in this country, which includes the assessment of genetic variation in annual [20] and perennial species [21, 22]. The major focus of this study was the evaluation of morphological characters and assessment of taxonomic position of *O. almaatensis* based on the use of the nuclear ribosomal DNA internal transcribed spacer region (ITS) [23]. ITS is a highly polymorphic DNA marker and widely recognized as a valuable tool in plant evolutionary studies [24–26]. The research is part of the new nation-wide research project [27] that combine efforts of local botanists and geneticists from Biotechnology Research Organizations, Botanical Gardens, National Nature Parks and Reserves.

## Methods

### Plant materials

Two populations of *Oxytropis almaatensis,* each containing twenty plants per population, were collected in 2015–2016 in two neighboring Gorges of Trans Ili Alatau Mountains. The plant leaves were collected randomly and dried in silica gel. GPS coordinates of two collected populations are given in Table 1.

**Table 1:** Geographical locations of two collected populations of *O. almaatensis*

Populations	Location	Altitude (m)	Coordinates (N)	Coordinates (E)
Population 1	Trans Ili Alatau mountains, Big Almaty gorge	2158–2160	43^0^04.864’	076^0^59.604’
Population 2	Trans Ili Alatau mountains, Small Almaty gorge	2012–2055	43^0^08.490’	077^0^04.198’

**Fig. 1:**
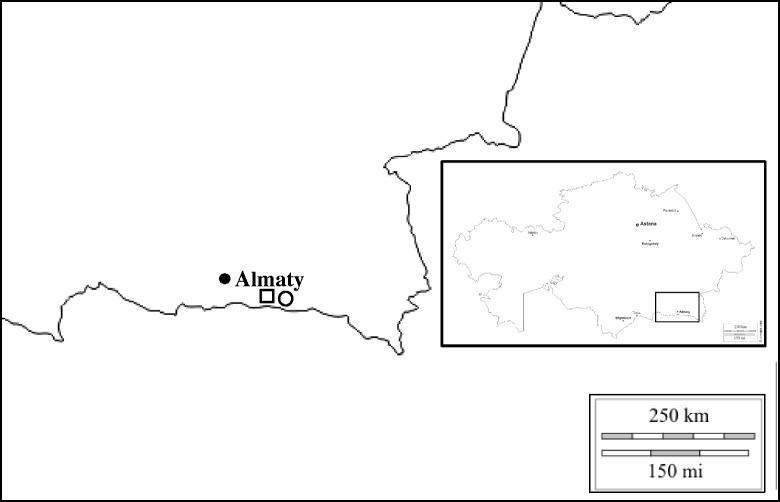
Geographic locations of two populations of *O. almaatensis* in the southeast region of Kazakhstan (square - Big Almaty gorge, population 1, circle - Small Almaty gorge, population 2)

Big Almaty and Small Almaty Gorges are located in Trans Ili Alatau mountain territory. The approximate locations of populations are shown in Fig. 1. They are growing approximately 10 km apart.

Trans Ili Alatau mountains is in the northern part of the Tian-Shan mountains system. The North Tian-Shan has a rich and unique flora, different from other regions of Kazakhstan. During the research process, the populations of *O. almaatensis* have been described in geobotanical and floristic aspects according to [28]. The nomenclature of plants was given according to Abdulina [16].

### Molecular work

Total genomic DNA was extracted using a modified CTAB protocol [29], which is detailed in Sramko et al. [30]. The internal transcribed spacers 1 and 2 (ITS) and the 5.8S rRNA gene [31] were PCR amplified. The PCR primers are given in Table 2. The ITS amplification was performed using Veriti Thermo cycler (Applied Biosystems, Foster City, CA, USA). PCR products were verified in 1.5% agarose gels. Single bands with expected sizes around 650 bp for ITS were cut out from gels and purified using ULTRAPrep® Agarose Gel Extraction Mini Prep Kit (AHN Biotechnologie GmbH, Nordhausen, Germany) according to the protocol provided by the company.

**Table 2:** Nucleotide sequences of the ITS primers [31]

Primers	Primer sequence	Annealing temperature
ITS1nF	5′-AGAAGTCGTAACAAGGTTTCCGTAGG- 3′ 5′-	58 °C
ITS4nR	TCCTCCGCTTATTGATATGC- 3′	

Purified DNA amplicons were used for the sequence reactions with forward and reverse primers separately. Ethanol/EDTA method [32] was used in precipitation of products. All reactions were performed with the BigDye Terminator Cycle Sequencing technology (Applied Biosystems, Foster City, CA, USA). Each reaction was carried out in 20 μl volume in concentrations described by the company. Sequence analysis was conducted on an ABI 3130 (Applied Biosystems, ThermoFisher Scientific, Waltham, MA, USA) DNA sequencer.

### Data analysis

A phylogenetic analysis was conducted using Maximum-Likelihood (ML) [33] in MEGA 5 [34] based on the ITS sequence alignment with 1000 bootstrap replications. The set of sequences included 26 *Oxytropis* species, including two samples of *O. almaatensis,* 5 samples of *O. glabra* (from NCBI) and two outgroup species *Astragalus mongholicus* and *Caragana dasyphylla* (Additional file 1). The names of the sections and subgenera are given according to the Malyshev [1].

## Results

### Description of sampling location and morphology of O. Almaatensis

During collecting trips in 2015–2016 two populations of *O. almaatensis* were found and sampled. Vouchers for herbarium specimens were provided to the department of biodiversity and bioresources of al-Farabi Kazakh National University. Each population consisted of 2 subpopulations and was divided based on differences in floristic composition (see descriptions below). The first population was located at an altitude 2158–2160 m on the western exposed slope in the spruce zone of the Big Almaty gorge of Trans-Ili Alatau mountains from the river side Kumbelsu. The dominant plant species in floristic composition of subpopulation 1 in decreasing number of plants were *Festuca valesiaca, Festuca rubra, Phleum phleoides, Oxytropis almaatensis, Geranium collinum, Thymus marschallianus, Ziziphora interupta, Origanum vulgare,* and *Achillea millefolium*; while in subpopulation 2 they were *Aquilegia atrovinosa, Sisymbrium brassiciform, Hypericum perforatum, Oxytropis almaatensis, Elymus tschimganicus*, *Poa stepposa, Festuca valesiaca, Calamagrostis pavlovii,* and *Alfredia nivea*.

The second population of *O. almaatensis* was found in the Small Almaty Gorge in the Sary-Say tract at an altitude 2012–2055 m on the slopes of south-west and north-west exposition of the right bank of the river Malaya Almatinka. The composition of the plant communities represented by following dominant plant species for subpopulation 3 in decreasing number of plants were *Eremurus robustus, Polygonum soongoricus, Dianthus tianschanicus, Erysimum transilensis, Oxytropis almaatensis, Festuca sulcata, Koeleria gracilis*, *Poa stepposa, Poa nemaralis,* and *Dactilis glomerata*; while for subpopulation 4 those species were *Ferula kelleri, Ferula akitschenkensis, Echium italicum, Nepeta panonica, Ziziphora bungeana, Verbascum thapsus, Hypericum perforatum, Festuca sulcata, Poa stepposa, Phleum phleoides,* and *Dactilis glomerata.*

Both populations of *O. almaatensis* were located on screes and on stony gravel deposits along the river and occupied small areas. The status of *O. almaatensis* populations, based on number of individuals in different age stages, showed that they are in fairly good condition, and there are no immediate threats to their extinction.

The morphological analysis of *O. almaatensis* using plants from the two collected populations, in the same generative adult phase, allowed the assessment of their similarities and differences. Twenty-six plant samples were used for the population 1 and 45 samples for population 2. The morphological analysis was based on several key traits such as plant height, leaf number, leaf length, pair leaflets number, leaflet length, leaflets width, peduncle number, peduncle length, flower number per one peduncle (Table 3). The t-test indicated that both populations were similar for plant height and peduncles number per plant. However, the populations were moderately different for pair leaflet numbers (*P* < 0.05) and peduncle length (*P* < 0.01), and highly different for traits such as leaf number, leaf length, leaflet length and width, and flower number per plant (*P* < 0.0001). In all traits, except in the trait “pair leaflet number”, traits in population 1 were larger than in population 2 (Table 3) showing the robustness of population 1 plants. Morphological comparison of *O. almaatensis* and *O. glabra* based on quantification of peduncle length to leaf length ratio confirmed clear differences between the two species. This ratio in *O. almaatensis* was 1.56 versus 1.0 in *O. glabra* (Additional file 2)*.*

**Table 3:** Biometrics of the generative individuals of *O. almaatensis* in different population

Pop	Studied samples	Plant height, cm	Leaf number, pcs.	Leaf length, cm	Pair leaflets number, pcs.	Leaflet length, cm	Leaflets width, cm	Peduncle number, pcs.	Peduncle length, cm	Flower number for one peduncle, pcs.	1000 seeds weight, g
1	26	26.35 ± 1.62.	30 ± 2.99	17.99 ± 0.58	15.61 ± 0.49	1.38 ± 0.04	0.54 ± 0.02	4.96 ± 0.46	27.34 ± 1.38	14.33 ± 0.68	3.8 ± 0.2
2	45	23.58 ± 0.9	13.58 ± 0.98	14.42 ± 0.46	17.51 ± 0.48	1.11 ± 0.04	0.42 ± 0.01	4.09 ± 0.33	22.83 ± 0.8	5.38 ± 0.52	3.7 ± 0.2
Mean		24.59 ± 0.83	19.59 ± 1.56	15.73 ± 0.41	16.81 ± 0.37	1.21 ± 0.03	0.47 ± 0.01	4.41 ± 0.27	24.48 ± 0.76	8.71 ± 0.66	3.75 ± 0.2
T-test		Not significant	*P* < 0.0001	*P* < 0.0001	*P* < 0.01	*P* < 0.0001	*P* < 0.0001	Not significant	*P* < 0.003	*P* < 0.0001	Not significant

### Assessment of the phylogenetic position of *O. almaatensis*

The phylogenetic tree included nrDNA (ITS) sequences of 26 *Oxytropis* species. A total of five samples of *O. almaatensis* from each populations were sequenced and were found to be identical for their ITS region. Therefore, only one sequence from each population of *O. almaatensis* was utilized in the alignment with 28 additional sequences of *Oxytropis* species and the outgroup samples of *Astragalus* and *Caragana* obtained from GenBank (Additional file 1).

**Fig. 2:**
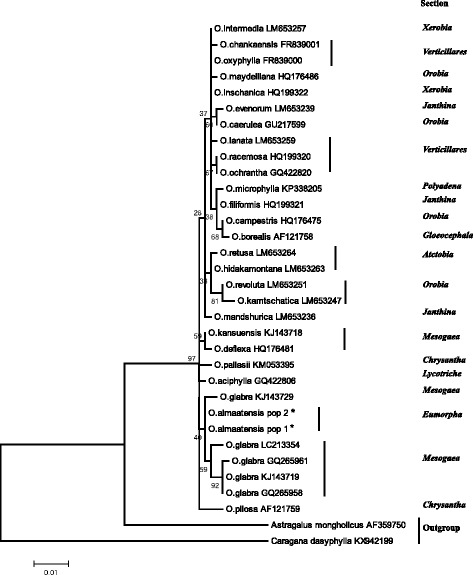
Phylogenetic tree for *Oxytropis* species based on ITS sequences using the Maximum Likelihood method. Taxonomical classification is given according to Malyshev [1]. Numbers at nodes show bootstrap values (%) an * indicates *O. almaatensis* collected in Kazakhstan. The sequences of *Astragalus mongholicus* and *Caragana dasyphylla* are used as a outgroup species

The ML phylogenetic tree for *Oxytropis* representatives is shown in Fig. 2. The tree shows the relatedness of *O. almaatensis* to *O. aciphylla, O. pilosa* and *O. deflexa,* but separated *O. almaatensis* and *O. glabra* into a distinct subclade from these species. The aligned length of the ITS region was 601 bp using only *Oxytropis* species, but the aligned length increased to 614 bp when the outgroup species (*Astragalus mongholicus, Caragana dasyphylla*) were included. The number of polymorphic sites without outgroup was 34 or 5.6% (Table 4). The largest number of polymorphic sites was found in ITS1 (21), followed by ITS2 (11) with only one variable site detected in 5.8RNA (coding) region (Table 4). The ITS sequence analysis in the alignment revealed that three polymorphic sites separate *O. almaatensis* and *O. glabra* (Table 4).

**Table 4:** Polymorphic sites of *Oxytropis* species based on alignment of ITS sequences for 26 *Oxytropis* species

№	ITS 1																					5.8S	ITS 2										
	1	2	3	4	5	6	7	8	9	10	11	12	13	14	15	16	17	18	19	20	21	23	24	25	26	27	28	29	30	31	32	33	34
Nucleotide position	26	28	45	57	60	66	67	68	76	82	90	108	116	118	121	123	124	167	180	203	204	393	419	420	422	426	438	469	495	496	508	561	568
*O.revoluta* LM653251	C	T	A	C	G	C	G	G	G	C	A	C	C	C	T	C	C	C	C	C	C	C	C	T	C	A	T	G	C	C	A	T	C
*O.retusa* LM653264	.	.	.	.	.	.	.	.	A	.	.	.	.	.	.	.	.	.	.	.	.	.	.	.	T	.	.	.	T	.	.	.	.
*O.racemosa* HQ199320	.	.	.	.	.	.	.	.	.	.	.	.	.	.	G	.	.	.	.	.	.	.	.	.	T	.	A	.	T	T	.	.	.
*O.pilosa* AF121759	.	.	.	.	A	.	.	.	.	.	.	.	.	T	G	.	.	.	.	.	.	.	.	C	T	.	.	.	T	.	G	C	.
*O.pallasii* KM053395	T	.	.	.	.	T	.	.	.	.	.	.	.	.	G	.	.	.	.	.	.	.	.	.	T	.	.	.	T	.	.	C	.
*O.oxyphylla* FR839000	.	.	.	.	.	.	.	.	.	.	.	.	.	.	G	.	.	.	.	.	.	.	.	.	T	.	A	.	T	.	.	.	.
*O.ochrantha* GQ422820	.	.	.	.	.	.	.	.	.	.	.	.	.	.	G	.	.	.	.	.	.	.	.	.	T	.	A	.	T	T	.	.	.
*O.microphylla* KP338205	.	.	.	.	.	.	.	.	.	.	.	.	.	.	.	T	.	.	.	.	.	.	.	.	T	.	A	.	T	.	.	.	.
*O.maydelliana* HQ176486	.	C	.	.	.	.	.	.	.	.	.	.	.	.	G	.	.	.	.	.	.	.	.	.	T	.	A	.	T	.	.	.	.
*O.mandshurica* LM653236	.	.	.	.	.	.	.	.	.	.	T	.	.	.	G	.	.	.	.	.	.	.	.	.	T	.	.	.	T	.	.	.	.
*O.lanata* LM653259	.	.	.	.	.	.	.	.	.	.	.	.	.	.	G	.	.	.	.	.	.	.	T	.	T	.	A	.	T	.	.	.	.
*O.kansuensis* KJ143718	.	.	.	.	.	.	.	.	.	.	.	.	.	.	G	.	.	.	.	T	.	.	.	.	T	.	.	.	T	.	.	C	.
*O.kamtschatica* LM653247	.	.	.	.	.	.	.	.	.	.	.	.	.	.	.	.	.	.	.	.	.	.	.	.	.	.	.	A	.	.	.	.	T
*O.intermedia* LM653257	.	.	.	.	.	.	.	.	.	.	.	.	.	.	G	.	.	.	.	.	.	.	.	.	T	.	A	.	T	.	.	.	.
*O.inschanica* HQ199322	.	.	.	.	.	.	.	.	.	.	.	.	.	.	G	.	.	.	.	.	.	.	.	.	T	.	A	.	T	.	.	.	.
*O.hidakamontana* LM653263	.	.	.	.	.	.	.	.	.	.	.	.	.	.	.	.	.	.	.	.	.	.	.	.	T	.	.	.	T	.	.	.	.
*O.filiformis* HQ199321	.	.	.	.	.	.	.	.	.	.	.	.	.	.	.	.	.	.	.	.	.	.	.	.	T	.	A	.	T	.	.	.	.
*O.evenorum* LM653239	.	.	.	.	.	.	.	C	.	.	.	.	.	.	G	.	T	.	.	.	.	.	.	.	T	.	A	.	T	.	.	.	.
*O.deflexa* HQ176481	.	.	.	.	.	.	.	.	.	.	.	.	.	.	G	.	.	.	.	T	.	.	.	.	T	.	.	.	T	.	.	C	T
*O.chankaensis* FR839001	.	.	.	.	.	.	.	.	.	.	.	.	.	.	G	.	.	.	T	.	.	.	.	.	T	.	A	.	T	.	.	.	.
*O.campestris* HQ176475	.	.	.	.	.	.	.	.	.	.	.	.	.	.	.	.	.	T	.	.	.	.	.	.	T	.	A	.	T	.	.	.	.
*O.caerulea* GU217599	.	.	.	.	.	.	.	C	.	.	.	.	.	.	G	.	.	.	.	.	.	.	.	.	T	.	A	.	T	.	.	.	.
*O.borealis* AF121758	.	.	.	.	.	.	.	.	.	.	.	.	.	.	.	.	.	T	.	.	.	G	.	.	T	.	A	.	T	.	.	.	.
*O.aciphylla* GQ422806	.	.	.	.	.	.	.	.	.	.	.	.	.	.	G	.	.	.	.	.	.	.	.	.	T	.	.	T	T	.	.	C	.
*O.almaatensis*	.	.	.	T	.	.	.	.	.	.	.	.	.	.	G	.	.	.	.	.	T	.	.	.	T	.	.	.	T	.	.	C	.
*O.glabra* LC213354	.	.	T	T	.	.	.	.	.	.	.	.	.	.	G	.	T	.	.	.	T	.	.	.	T	G	.	.	T	.	.	C	.
*O.glabra* KJ143729	T	.	.	T	.	.	.	.	.	.	.	T	.	.	G	.	.	.	.	.	T	.	.	.	T	.	.	.	T	.	.	C	.
*O.glabra* KJ143719	.	.	.	T	.	.	T	.	.	.	.	.	A	.	G	.	.	.	.	.	T	.	.	.	T	G	.	.	T	.	.	C	.
*O.glabra* GQ265958	.	.	.	T	.	.	T	.	.	.	.	.	A	.	G	.	.	.	.	.	T	.	.	.	T	G	.	.	T	.	.	C	.
*O.glabra* GQ265961	.	.	.	T	.	.	T	.	.	T	.	.	A	.	G	.	.	.	.	.	T	.	.	.	T	G	.	.	T	.	.	C	.

## Discussion

In this study, we attempted to clarify the systematic position of *O. almaatensis,* which is a narrow endemic species of the Trans Ili Alatau mountains. Based on botanical descriptions, *O. almaatensis* was previously classified in section *Eumorpha* Bunge of subgenera *Euoxytropis* (Boiss.) Bunge [16]. Later, Malyshev [1] classified this section as part of the subgenus *Oxytropis*, represented by species that occur in the mountains of Central Asia. Ten samples collected from each of two different populations (10 total samples) of *O. almaatensis* showed no variation in their ITS sequences. The phylogenetic tree based on ITS sequences clearly grouped *O. almaatensis* and *O. glabra* together (Fig. 2). It should be noted that classical morphological classification placed these taxa in different sections: *Eumorpha* and *Mesogeae*, respectively. One of the main differences between these two sections is presence of stems in *Mesogea*, whereas *Eumorpha* has stemless species. However, several other traits show high similarities between the two species. The morphological data for *O. almaatensis* (in this study) and literature data for *O. glabra* [18, 35] indicated that the shape and size of leaves, and paired leaflets are very similar (Additional file 2). In addition, the species share similar habitats in the Tien Shan mountains [18]. The topology of the ML tree suggests that *Oxytropis* has a monophyletic origin and confirms previous reports with similar results [10, 13, 14]. The analysis of the polymorphic sites of the ITS showed that 34 out of 614 nucleotides were variable (Table 4) for ITS1, 5.8RNA, and ITS2, and eight polymorphic nucleotides in positions 26, 45, 67, 82, 108, 116, 124 and 426 separated *O. almaatensis* from *O. glabra.* Recently, two papers [12, 26] have discussed the level of variability in different DNA barcoding markers for differentiation of medicinal plants. The authors suggested that ITS2 is one of the most appropriate markers in species identification and has a number of advantages over other types of markers, including ITS1. However, results in this study indicated that the number of polymorphic sites in ITS1 was higher (21) than in ITS2 (11) (Table 4).

As shown in a number of reports [8, 13, 14], the taxonomy of *Oxytropis* is very complicated because of contradictory results for the positioning of species into particular sections and subgenera based on classical botanical studies. The results obtained from the molecular taxonomic studies [10, 13, 14] were also not congruent because different studies employed different species and different types of DNA markers. Understandably, molecular phylogenetic trees often did not match those proposed by classical morphological studies. The results from the present study is not much different from previously published results in that two species (*O. almaatensis* and *O. glabra*), from two different sections, were grouped together in the same subclade. However, many key morphological traits used for the differentiation of sections may rely on single or few genes and may not be associated with analyses of markers from non-coding regions of the genome. Therefore, in order to appreciate the wealth of phenotypic interspecies and intra-specific differences, such as discovered in two populations of *O. almaatensis,* it is critical to apply modern genomic technologies, including next-generation sequencing approaches to a very large and representative set of *Oxytropis* taxa to infer reliably the taxonomic position of species within the genus.

## Conclusions

The alignment of ITS sequences from samples of two populations of *O. almaatensis* revealed all the sequences were identical. The ML phylogenetic tree based on ITS sequences showed that *O. almaatensis* and *O. glabra* were separated into a distinct subclade. The topology of the tree confirmed the monophyletic origin of *Oxytropis*. The analysis of the ITS nucleotide alignment revealed three polymorphic sites between *O. almaatensis* and *O. glabra.* The variability of ITS1 was higher (21 polymorphic sites) than in the ITS2 region (11). Morphological analysis of individual plants from two populations collected in neighboring gorges of Trans-Ili Alatau Mountains in Kazakhstan showed a specific pattern in phenotypic variation. The two populations were similar in plant height and peduncles number per plant, but significantly different for traits such as leaf number, leaf length, leaflet length and width, and flower number per plant (*P* < 0.0001).

## Additional files

Additional file 1: Accession number of species obtained from NCBI. (DOCX 15 kb)

Additional file 2: Comparative morphological description of *O.almaatensis* and *O.glabra*. (DOCX 14 kb)
