# Supplementary material for: Phylogenetic study of the endemic species Oxytropis almaatensis (Fabaceae) based on nuclear ribosomal DNA ITS sequences
Source: BMC Plant Biol. 2017 Nov 14;17(Suppl 1):173. doi: 10.1186/s12870-017-1128-x (PMC5688500; doi:10.1186/s12870-017-1128-x)
Supplement: Supplementary file 1 — Accession number of species obtained from NCBI. (DOCX 15 kb) [file 12870_2017_1128_MOESM1_ESM.docx]

| **Table S1 Accession number of species obtained from NCBI** | |
| --- | --- |
| **NCBI accession number** | **Species** |
| LM653251 | *Oxytropis revoluta* |
| LM653264 | *Oxytropis retusa* |
| HQ199320 | *Oxytropis racemosa* |
| AF121759 | *Oxytropis pilosa* |
| KM053395 | *Oxytropis pallasii* |
| FR839000 | *Oxytropis oxyphylla* |
| GQ422820 | *Oxytropis ochrantha* |
| KP338205 | *Oxytropis microphylla* |
| HQ176486 | *Oxytropis maydelliana* |
| LM653236 | *Oxytropis mandshurica* |
| LM653259 | *Oxytropis lanata* |
| KJ143718 | *Oxytropis kansuensis* |
| LM653247 | *Oxytropis kamtschatica* |
| LM653257 | *Oxytropis intermedia* |
| HQ199322 | *Oxytropis inschanica* |
| LM653263 | *Oxytropis hidakamontana* |
| GQ265958 | *Oxytropis glabra* |
| LC213354 | *Oxytropis glabra* |
| KJ143729 | *Oxytropis glabra* |
| KJ143719 | *Oxytropis glabra* |
| GQ265961 | *Oxytropis glabra* |
| HQ199321 | *Oxytropis filiformis* |
| LM653239 | *Oxytropis evenorum* |
| HQ176481 | *Oxytropis deflexa* |
| FR839001 | *Oxytropis chankaensis* |
| HQ176475 | *Oxytropis campestris* |
| GU217599 | *Oxytropis caerulea* |
| AF121758 | *Oxytropis borealis* |
| GQ422806 | *Oxytropis aciphylla* |
| AF359750 | *Astragalus mongholicus* |
| KX942199 | *Caragana dasyphylla* |
