# Supplementary material for: Phylogenetic study of the endemic species Oxytropis almaatensis (Fabaceae) based on nuclear ribosomal DNA ITS sequences
Source: BMC Plant Biol. 2017 Nov 14;17(Suppl 1):173. doi: 10.1186/s12870-017-1128-x (PMC5688500; doi:10.1186/s12870-017-1128-x)
Supplement: Supplementary file 2 — Comparative morphological description of O.almaatensis and O.glabra. (DOCX 14 kb) [file 12870_2017_1128_MOESM2_ESM.docx]

**Table S2 Comparative morphological description of *O.almaatensis* and *O.glabra***

| \| **Traits** \| ***Oxytropis almaatensis***  (in this study) \| ***Oxytropis glabra***  (Flora of Kazakhstan, V.5, 1961) \| \| --- \| --- \| --- \| \| **Plant height, cm** \| 24.59±0.83 \| 5-70 \| \| **Leaves length, cm** \| 15.73±0.41 \| 2-10 \| \| **Leaflet length, cm** \| 1.21±0.03 \| 4-12 \| \| **Leaflets width, cm** \| 0.47±0.01 \| 0,2-0,4 \| \| **Pair leaflets number** \| 16.81±0.37 \| 7-12 \| \| **Peduncle length, cm** \| 24.48±0.76 \| Peduncles equal or slightly higher than leaves \| \| **Calyx shape** \| Tube campanulate \| Campanulate \| \| **Corolla color** \| Pink-purple \| Bluish purple \| \| **Bean length, mm** \| 18-20 \| 10-14 \| \| **Bean shape** \| Prolate-lanceolate \| Tightly webbed oblong \| \| **Blooming** \| May-June \| June-July \| \| **Fruitification** \| July-August \| July-September \| \| **Habitats** \| Trans Ili Alatau mountains (Tian Shan) \| Central Asia (Tian Shan, Pamir Alai), Western and Eastern Siberia, Mongolia \| |
| --- | --- | --- | --- | --- | --- | --- | --- | --- | --- | --- | --- | --- | --- | --- | --- | --- | --- | --- | --- | --- | --- | --- | --- | --- | --- | --- | --- | --- | --- | --- | --- | --- | --- | --- | --- | --- | --- | --- | --- | --- | --- | --- |
|  |
